# The Treatment of Inebriety by Atropine

**Published:** 1904-07-16

**Authors:** 


### THE TREATMENT OF INEBRIETY BY ATROPINE.

Dr. C. A. McBride[1] reports a number of cases of inebriety treated by atropine and expresses the conviction that a complete recovery with absence of all craving for stimulants may generally be expected after a six weeks' course of treatment. Long periods of detention are in the majority of cases wholly unnecessary. Dr. McBride, in addition to atropine, uses strychnine and a fluid extract of cinchona bark. He insists upon the need for the personal supervision of the medical attendant, and allowing for peculiarities of individual cases, advises:—1. Atropine sulphate (hypodermically) $\frac{1}{200}$ gr. thrice daily, increased during the first week until the throat is dry and the pupils are affected; in most cases $\frac{1}{50}$ gr. is found to be the maximum dose. 2. Strychnine nitrate given at the same time as the atropine, and also hypodermically, $\frac{1}{60}$ gr. increased to $\frac{1}{20}$ gr. 3. Fluid extract of red cinchona every three hours by the mouth. Warm baths, regulated exercises, and other measures affecting the general health are auxiliary influences of importance. Close personal attention is necessary on the part of the physician, and the treatment is hardly likely to be efficiently practised unless the patient is under the discipline of a sanatorium or other suitable institution.

[1] Brit. Med. Jour., April 30, 1904.

